# Modeling Klinefelter Syndrome Using Induced Pluripotent Stem Cells Reveals Impaired Germ Cell Differentiation

**DOI:** 10.3389/fcell.2020.567454

**Published:** 2020-10-07

**Authors:** Olivier Botman, Youssef Hibaoui, Maria G. Giudice, Jérôme Ambroise, Catherine Creppe, Anis Feki, Christine Wyns

**Affiliations:** ^1^Gynecology Unit, Institut de Recherche Expérimentale et Clinique (IREC), Université catholique de Louvain, Brussels, Belgium; ^2^Department of Gynecology-Andrology, Cliniques Universitaires Saint-Luc, Brussels, Belgium; ^3^Stem Cell Research Laboratory, Department of Obstetrics and Gynecology, Geneva University Hospitals, Geneva, Switzerland; ^4^Department of Obstetrics and Gynecology, Hôpital Fribourgeois (HFR) Fribourg, Hôpital Cantonal, Fribourg, Switzerland; ^5^Center for Applied Molecular Technologies (CTMA), Institut de Recherche Expérimentale et Clinique (IREC), Université catholique de Louvain, Brussels, Belgium; ^6^Groupe Interdisciplinaire de Génoprotéomique Appliquée (GIGA)-Signal Transduction, C.H.U. Sart Tilman, University of Liège, Liège, Belgium

**Keywords:** induced pluripotent stem cells, primordial germ cells, germ cell differentiation, post-meiotic cells, Klinefelter syndrome, Klinefelter syndrome iPSCs

## Abstract

Klinefelter syndrome (KS), with an incidence between 1/600 and 1/1,000, is the main genetic cause of male infertility. Due to the lack of an accurate study model, the detailed pathogenic mechanisms by which this X chromosome aneuploidy leads to KS features remain unknown. Here, we report the generation and characterization of induced pluripotent stem cells (iPSCs) derived from a patient with KS: 47XXY-iPSCs. In order to compare the potentials of both 47XXY-iPSCs and 46XY-iPSCs to differentiate into the germ cell lineage, we developed a directed differentiation protocol by testing different combinations of factors including bone morphogenetic protein 4 (BMP4), glial-derived neurotrophic factor (GDNF), retinoic acid (RA) and stem cell factor (SCF) for 42 days. Importantly, we found a reduced ability of 47XXY-iPSCs to differentiate into germ cells when compared to 46XY-iPSCs. In particular, upon germ cell differentiation of 47XXY-iPSCs, we found a reduced proportion of cells positive for BOLL, a protein required for germ cell development and spermatogenesis, as well as a reduced proportion of cells positive for MAGEA4, a spermatogonia marker. This reduced ability to generate germ cells was not associated with a decrease of proliferation of 47XXY-iPSC-derived cells but rather with an increase of cell death upon germ cell differentiation as revealed by an increase of LDH release and of capase-3 expression in 47XXY-iPSC-derived cells. Our study supports the idea that 47XXY-iPSCs provides an excellent *in vitro* model to unravel the pathophysiology and to design potential treatments for KS patients.

## Introduction

Klinefelter syndrome (KS), with an incidence between 1/600 and 1/1,000, is the main genetic cause of male infertility as KS represent 11% of azoospermic men and 4% of infertile men (Van Assche et al., 1996). The vast majority of KS patients (80–90%) are non-mosaic with a 47XXY karyotype whereas 10–20% of KS patients are mosaic forms of the disorder (with 47XXY and 46XY karyotype) or higher grade of aneuploidy (48XXXY karyotype) (Lanfranco et al., 2004; Gravholt et al., 2018). Histological analysis of testes from KS patients reveals an extensive fibrosis and hyalinization of the seminiferous tubules with a progressive decline in germ cells, starting before puberty, intensifying at puberty, and ultimately leading to azoospermia (Wikström et al., 2007). Sertoli cells (SCs) which support germ cells, not only decrease in number but also show impaired function with attenuated maturation (Aksglæde et al., 2011) while Leydig cells (LCs) exhibit hyperplasia (Bojesen et al., 2011). A dysfunctional somatic compartment with reduced expression of androgen receptor in SCs and of INSL3 in LCs, both as maturation markers was recently reported (Giudice et al., 2019). Although KS is associated with male infertility, the pathogenic process by which the extra copy of X chromosome leads to these defects remains unknown. Transgenic mouse models of KS have evidenced a decrease in primordial germ cell (PGC) populations during migration through the epiblastic crest (Mroz et al., 1999) providing a number of insights in KS pathogenesis. However, these models do not accurately recapitulate the human condition.

The discovery that human pluripotent stem cells (PSCs) can be reprogrammed from somatic cells to further differentiate into germ-like cells provided new opportunities to investigate germ cell development and function (Clark et al., 2004; Kee et al., 2009; Park et al., 2009; Panula et al., 2010; Eguizabal et al., 2011; Easley et al., 2012; Medrano et al., 2012; Ramathal et al., 2014; Mahabadi et al., 2018; Li et al., 2020). Also, these cells make excellent *in vitro* models, replicating disease-associated phenotypes (Hibaoui and Feki, 2012; Botman and Wyns, 2014). Recent studies have been successful in generating induced pluripotent stem cells from patients with KS (Ma et al., 2012; Shimizu et al., 2016; Panula et al., 2019). In the present study, we have generated iPSCs from a patient with KS: 47XXY-iPSC line#11 and 47XXY-iPSC line#16. A 46XY-iPSC line generated from a healthy individual was used as control (Grad et al., 2011; Hibaoui et al., 2014). We evaluated the multilineage potential of these iPSCs *in vivo* by teratoma formation when these iPSCs were injected intramuscularly into immunodeficient SCID mice. In order to study KS pathogenesis, we developed a germ cell differentiation protocol by testing different combinations of factors, including bone morphogenetic protein 4 (BMP4), glial-derived neurotrophic factor (GDNF), retinoic acid (RA), and stem cell factor (SCF) for 42 days. The potentials of both 47XXY-iPSCs and 46XY-iPSCs to differentiate into germ cell lineage was also investigated.

## Materials and Methods

### iPSC Derivation and Culture

Skin fibroblasts were isolated from a 20-years-old infertile KS patient. These 47XXY-fibroblasts were used to generate 47XXY-iPSCs by transducing the parental fibroblasts with the polycistronic lentiviral vector, carrying the pluripotent genes *OCT4*, *KLF4*, *SOX2*, and *c-MYC* as we previously described (Grad et al., 2011; Hibaoui et al., 2014). A 46XY-iPSC line derived from a healthy individual with the same method of reprogramming was used as a control (Grad et al., 2011; Hibaoui et al., 2014). Among the 47XXY-iPSC lines generated from the parental 47XXY-fibroblasts, 47XXY-iPSC line#11 and 47XXY-iPSC line#16 were used for the present study. Theses iPSC lines were cultured on primary human foreskin fibroblasts (iHFF 106-05n, ECACC Culture Collections Public Health England, Salisbury, United Kingdom) that were mitotically inactivated by irradiation at 25 Gy. They were maintained with daily changes in knockout (KO)-DMEM medium supplemented with 20% serum replacement, 2 mmol/L GlutaMAX, 50 U/mL penicillin, 50 mg/mL streptomycin, 100 μmol/L β-mercaptoethanol, 100 μmol/L non-essential amino acids (all from Life Technologies, Carlsbad CA, United States) and 100 ng/mL β-fibroblast growth factor (β-FGF from Prospec, Ness-Ziona, Israel). The cell lines were then passaged mechanically in the presence of 10 μM ROCK-inhibitor Y-27632 (Sigma-Aldrich, St. Louis, MO, United States). Alternatively, these iPSCs were maintained in feeder-free conditions, on matrigel-coated dishes in StemFlex medium supplemented with 50 U/mL penicillin and 50 mg/mL streptomycin (Life Technologies, Carlsbad CA, United States) with media changes every 2 days. All cell lines were kept at 37°C in 5% CO_2_.

### Spontaneous Differentiation Into Three Germ Layers

Whole iPSC colonies were collected and seeded onto ultra-low attachment dishes (Sigma-Aldrich, St Louis MO, United States) in KO-DMEM supplemented with 20% newborn calf serum, 2 mmol/L glutaMAX, 50 U/mL penicillin, 50 mg/mL streptomycin 1% non-essential amino acid (all from Life Technologies, Carlsbad CA, United States) and 0.1 mmol/L β-mercaptoethanol (Sigma-Aldrich, St Louis MO, United States). Within 24 h, the cells had aggregated into EBs. After 7 days of suspension, these EBs were seeded onto gelatin-coated glass slides for an additional 14 days to allow the cells to differentiate. Medium was changed every 2 days.

### Germ Cell Lineage Differentiation

The iPSC colonies were dissociated with cell dissociation medium (Sigma-Aldrich, St. Louis MO, United States), centrifuged for 5 min at 1,000 rpm and resuspended in iPSC proliferation medium containing 2 μM ROCK inhibitor Y-27632 to improve cell survival. Then, these cells were allowed to aggregate in Aggrewell^TM^ dishes for 24 h in order to obtain size-calibrated EBs containing 4,000 cells. These EBs were transferred into low attachment wells (Costar, Corning Life Sciences) for 5 days in differentiation medium supplemented or not with 20 ng/mL BMP4 (Life Technologies, Carlsbad CA, United States) as outlined in Figure 3A. Approximately 30 EBs (∼120,000 cells) were seeded onto a glass slide coated with gelatin in one well of a 4-well plate and 4 different media were used to culture them over 16 additional days, as shown in Figure 3A. For the following 14 days, only retinoic acid (RA) (2 μM, Sigma) was added, and before including stem cell factor (SCF) (100 ng/mL, Life Technologies) from day 35 to day 42. EBs were retrieved for characterization after 5, 21, and 42 days of differentiation (Figure 3A).

### Karyotyping

Karyotyping was performed on at least 20 metaphase spreads using the GTG-banding method. Briefly, iPSCs were incubated in culture medium, supplemented with 0.2 mg/mL colcemid (Roche, Bâle, Switzerland) at 37°C for 20 min and washed three times in 2 mL PBS containing Ca^2+^ and Mg^2+^ (Life Technologies, Carlsbad CA, United States). A minimum of 15 colonies were collected in 2 mL 1 × trypsin-EDTA (Life Technologies, Carlsbad CA, United States) and incubated at 37°C for 5 min. Trypsin activity was then stopped and the cells were centrifuged at 300 g for 10 min. The pellet obtained was resuspended and incubated in 1 mL pre-warmed potassium chloride solution (KCl, 0.075 M) for 10 min at 37°C. The cells were subsequently pre-fixed in 1 mL Carnoy fixative solution (methanol/acetic acid = 3/1) at −20°C before immediate centrifugation at 1,800 g for 10 min. Finally, the supernatant was discarded and the pellet was once again suspended in Carnoy fixative solution and prepared for analyses.

### Alkaline Phosphatase Staining

iPSC colonies were fixed in 4% paraformaldehyde in PBS and treated with 0.1% Triton X-100 in PBS. The colonies were stained with alkaline phosphatase solution as described in the alkaline phosphatase substrate kit III manual (Vector Laboratories Ltd., Peterborough, United Kingdom) for 30–45 min at 37°C.

### Immunohistochemistry

Briefly, iPSCs and embryoïd bodies were fixed in 4% paraformaldehyde in phosphate buffered saline (PBS) for 30 min, permeabilized with 0.2% Triton X-100 for 30 min, and blocked with 5% bovine serum albumin in PBS for 1 h at room temperature (RT). Cells were incubated with primary antibody overnight at 4°C, washed with PBS and incubated with secondary antibody for 1 h at RT. The antibodies used for immunohistochemical staining are listed in Supplementary Table S1. The cells were finally with Vectashield mounting medium containing DAPI for nuclei identification. Images of immunostained cells were captured on a Mirax Midi fluorescence scanner (Zeiss MicroImaging GmbH, Jena, Germany) in automation. All immunostaining analyses were performed at least in triplicate and analyzed with ImageJ software (National Institutes of Health). For negative controls, primary antibodies were replaced with corresponding immunoglobulin serotypes. The proportion of positive cells was calculated by the difference observed between cells positive for primary antibodies and total cells identified by DAPI. All counting results were expressed as a percentage.

### Cytotoxicity Assays

Cell damage was evaluated by measuring lactate dehydrogenase (LDH) release into the medium by means of a UV assays on a Cobas Integra analyzer using the LDHI2 kit (Roche, Bâle, Switzerland). Briefly, 4 μL of supernatant was pipetted at different time points in iPSC differentiation (5, 21, 35, and 42 days) in each condition. Sample absorbance was measured at 340/659 nm and the results were expressed in IU/L.

### RNA Extraction, Non-quantitative, and Quantitative Real Time Polymerase Chain Reaction

Total RNA was extracted from iPSC-derived cells (2 wells of a 4-well plate were pooled) using the Qiagen RNeasy mini kit (Qiagen, Venlo, Netherlands) according to the manufacturer’s protocol. RNA quantities were assessed with a Nanodrop 2000 analyzer (Thermo Fisher Scientific, Waltham, MA, United States) and RNA integrity Number (RIN) analyses were conducted with the Agilent RNA 6000 nano kit (Agilent Technologies, Santa Clara, United States). Five-Hundred nanograms of RNA was reversed-transcribed with ThermoScript reverse transcriptase following the manufacturer’s instructions (Life Technologies, Carlsbad CA, United States). cDNA was amplified by real-time polymerase chain reaction (RT-PCR) in a Light Cycler 480 (Roche) using the Power SYBRGreen PCR master mix (Life Technologies, Carlsbad CA, United States). For amplification, the program used was 50°C for 2 min, 95°C for 10 min, 40 cycles of 95°C for 15 s and 60°C for 1 min. A mean quantity was calculated from duplicate PCR reactions for each sample, and this quantity was normalized against the *GAPDH* housekeeping gene. Each PCR reaction was performed at least in triplicate with negative controls and mean quantities were calculated in each case. For non-quantitative PCR, reactions were performed in a Biometra thermocycler (Göttingen, Germany), using the above program of amplification, with a RedTaq polymerase mix (Sigma-Aldrich, St. Louis, MO, United States), 250 nM primers and 1 μL of cDNA. Primer sequences used for non-quantitative and quantitative RT-PCR are listed in Supplementary Tables S2, S3.

### Teratoma Formation Assays

*In vivo* differentiation was investigated by teratoma formation as previously described (Hibaoui et al., 2014). All the procedures involving animals were conducted in accordance with the Swiss Federal Veterinary Office’s guidelines, based on the Swiss Federal Law on Animal Welfare and approved by the Ethics Review Board and the Committee on Animal Research of the Catholic University of Louvain. Briefly, 5 × 10^6^ cells were harvested from each iPSC and injected intramuscularly into SCID mice. After 8 weeks, the resulting teratomas were excised, fixed in 4% formaldehyde, and embedded in paraffin for immunohistochemistry analysis with horseradish peroxidase (HRP) using the Ventana Discovery automated staining system (Ventana Medical Systems, Tucson, AZ, United States). Ventana reagents were utilized for the entire procedure. No antigen retrieval pretreatment was required for either SMA or AFP antibodies. After automatic deparaffinization, slides were incubated for 30 min at 37°C SMA 1/300 (M0851, Dako, Baar, Switzerland) and AFP 1/750 (NCL-AFPp, Novocastra Laboratories Ltd, Newcastle upon Tyne, United Kingdom) with primary antibodies in antibody diluent from Dako (S2022; Glostrup, Denmark). For nestin antigen retrieval, the section was heated in CC1 cell conditioning solution for 36 min (EDTA antigen retrieval solution pH 8.4) using a standard protocol. Anti-nestin antibodies (MAB1259, R&D systems, Inc.) were applied at a dilution of 1/1,000 and also incubated for 30 min at 37°C. Detection of primary antibodies was carried out using secondary universal biotinylated antibody reagent and the amplified DAB map kit (Ventana Medical Systems, Tucson, AZ, United States), based on conversion of diaminobenzidine to a dye with multimeric HRP. For negative controls, AFP antibodies were replaced with rabbit IgG and SMA and nestin antibodies were replaced by mouse IgG serotypes.

### Statistical Analysis

For all experiments, comparisons between groups were conducted using regression models taking into account the effect of treatment and of cell lines, with 46XY-iPSCs serving as a reference. For some outcomes, log transformation was applied in order to satisfy assumptions of normality. *P* ≤ 0.05 were considered significant. When the treatment effect was evaluated on multiple outcomes, the Benjamini-Hochberg correction procedure was used to correct *p*-values (convert to *q*-values) and maintain a false discovery rate of 0.05. All statistical analyses were performed with R statistical software (version 3.1.2, R Statistical Computing, Vienna) and data are presented in bar and scatterplot graphs showing variance around the mean according to the new standard of data presentation (Weissgerber et al., 2015).

### Ethics Statement

Skin fibroblasts were isolated from patients after obtaining written informed consent with the approval of the ethics committee of the Cliniques Universitaires Saint-Luc.

## Results

### Generation and Characterization of 47XXY-iPSCs

**FIGURE 1:**
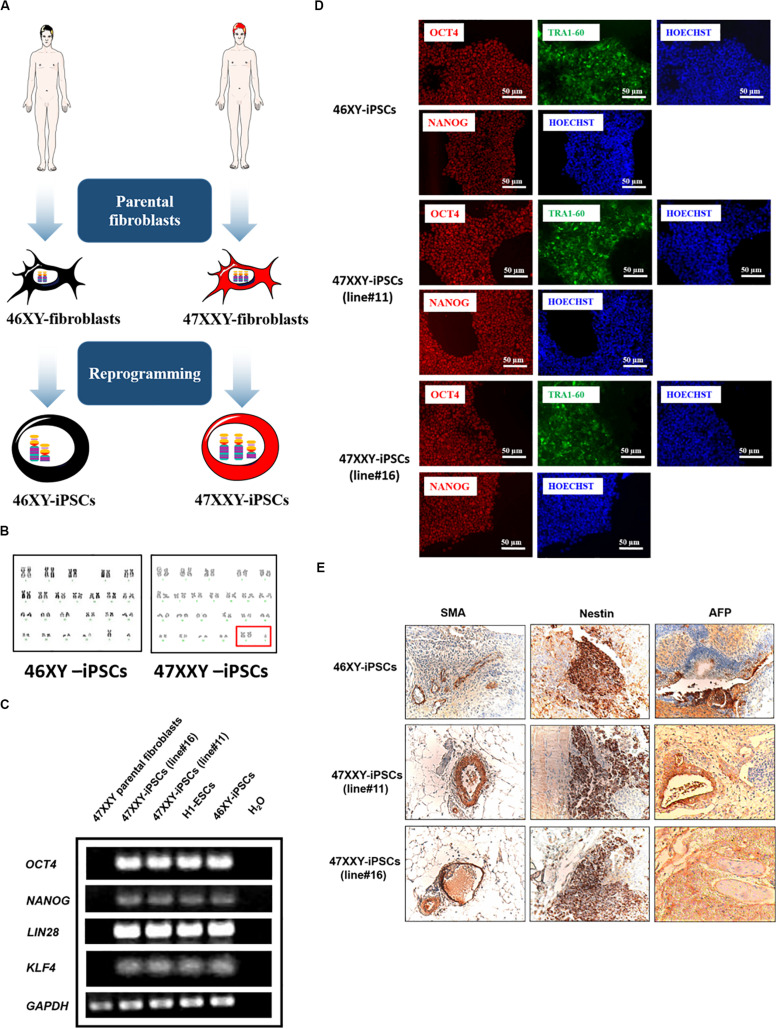
46XX-iPSCs and 47XXY-iPSCs exhibit markers of pluripotency. **(A)** Schematic representation for the reprogramming of 46XY and 47XXY parental fibroblasts into 46XY-iPSCs and 47XXY-iPSCs using *OCT4*, *SOX2*, *KLF4*, and *c-MYC* genes **(B)** Karyotypes of 46XY-iPSCs and 47XXY-iPSCs are 46, XY and 47, XXY, respectively. **(C)** RT-PCR of pluripotency-related genes *NANOG*, *OCT4*, *KLF4*, and *LIN28* in 46XY-iPSCs and 47XXY-iPSCs. The human embryonic stem cell line H1 (H1-ESCs) and the 47XXY parental fibroblasts were used as positive and negative controls for *OCT4*, *NANOG*, *KLF4*, and *LIN28* expression, respectively. **(D)** Immunofluorescence staining of 46XY-iPSC and 47XXY-iPSC lines for the pluripotency markers NANOG, OCT4 and TRA1-60. **(E)** Immunohistochemistry analysis of teratoma sections generated after intramuscular injection of 46XY-iPSC and 47XXY-iPSC lines into SCID mice. These teratomas expressed α-SMA (mesoderm), AFP (endoderm) and β3-tubulin (ectoderm).

47XXY-fibroblasts were isolated from a patient with Klinefelter syndrome (KS) and used to establish 47XXY-iPSCs using *OCT4*, *SOX2*, *KLF4*, and *c-MYC* genes as we previously described (Grad et al., 2011; Hibaoui et al., 2014). Among the 47XXY-iPSC lines generated from the parental 47XXY-fibroblasts, 47XXY-iPSC line#11 and 47XXY-iPSC line#16 were used for the present study. A 46XY-iPSC line generated with the same method of reprogramming was used as a control line (Figure 1A; Grad et al., 2011; Hibaoui et al., 2014). Then, these iPSCs were evaluated to confirm the genotype of the parental somatic cells. As revealed by GTG banding analysis, the 47XXY-iPSC lines showed a supernumerary X chromosome while 46XY-iPSCs had a normal karyotype (Figure 1B). RT-PCR analysis demonstrated expression of endogenous pluripotent transcription factors including *OCT4*, *NANOG*, *KLF4*, and *LIN28* in the generated 47XXY-iPSC lines in contrast with their parental fibroblasts (Figure 1C). This analysis also confirmed the expression of *OCT4*, *NANOG*, *KLF4*, and *LIN28* in the 46XY-iPSCs as in the human embryonic stem cell line H1 (H1-ESCs) (Figure 1C). As expected for cells that have acquired a pluripotent state, transgene silencing of exogenous factors after expansion of approximately ten passages of 47XXY-iPSC lines was demonstrated by RT-PCR (Supplementary Figure S1A). In line with this, all iPSC lines expressed markers of pluripotent stem cells including OCT4, NANOG, and TRA1-60 (Figure 1D) and showed alkaline phosphatase activity (Supplementary Figure S1B).

**FIGURE 2:**
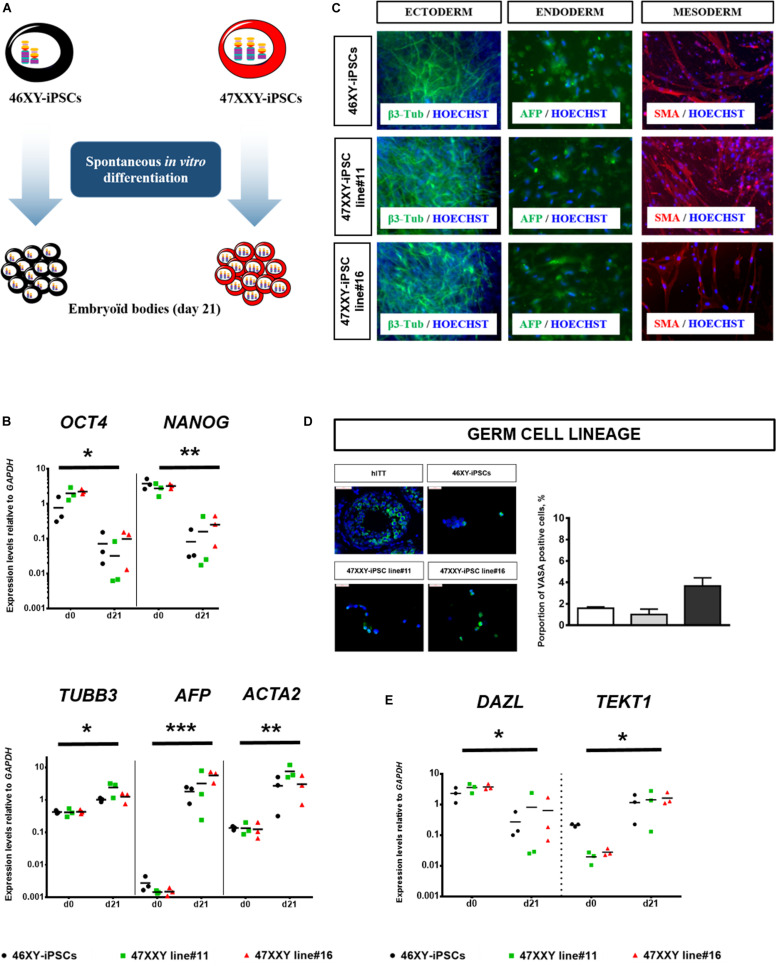
Spontaneous *in vitro* differentiation of 46XX-iPSCs and 47XXY-iPSCs. **(A)** Schematic representation for spontaneous *in vitro* differentiation of 46XY-iPSCs and 47XXY-iPSCs into the three embryonic germ layers as embryoïd bodies (EBs) in suspension culture for 4 days and as adherent cells for an additional 17 days. **(B)** Quantitative RT-PCR of pluripotency-related genes (*NANOG* and *OCT4*) and of markers for mesoderm (*ACTA2*), endoderm (*AFP*), and ectoderm (*TUBB3*). Expression levels are expressed relative to *GAPDH*. **(C)** Immunofluorescence staining of 46XY-iPSC and 47XXY-iPSC lines upon spontaneous *in vitro* differentiation, for markers of mesoderm (α-SMA), endoderm (AFP) and ectoderm (β3-tubulin). **(D)** Immunofluorescence staining of 46XY-iPSC and 47XXY-iPSC lines upon spontaneous *in vitro* differentiation for the germ cell marker VASA. Quantitative analysis of the proportion of VASA positive cells after 21 days of spontaneous *in vitro* differentiation. Human immature testicular tissue (hITT) was used as a positive control (scale = 50 μM). **(E)** Quantitative RT-PCR analysis of markers of germ cell development (*DAZL* and *TEKT1*) upon spontaneous *in vitro* differentiation of 46XY-iPSC and 47XXY-iPSC lines. Expression levels are expressed relative to *GAPDH*. Data are represented as variance around mean, ^∗^*p* < 0.05, ^∗∗^*p* < 0.01 and ^***^p < 0.001 between day 0 and day 21 from *n* = 3 independent experiments.

Moreover, both 47XXY-iPSCs and 46XY-iPSCs were evaluated for their developmental potential *in vivo* by injecting these iPSCs intramuscularly into immunodeficient SCID mice. Immunohistochemistry analysis revealed that these iPSC lines formed teratoma with all embryonic germ layers as detected by the expression of the ectodermal marker β3-tubulin, the mesodermal marker α-smooth muscle actin (α-SMA) and the endodermal marker α-fetoprotein (AFP) (Figure 1E). 47XXY-iPSCs and 46XY-iPSCs were also characterized to confirm their multi-lineage differentiation potentials *in vitro* into embryoïd bodies (EBs) (Figure 2A). As expected, upon 3 weeks of spontaneous *in vitro* differentiation, both 47XXY-iPSCs and 46XY-iPSCs exhibited a decreased expression of the pluripotent genes *OCT4* and *NANOG* (Figure 2B). Concomitantly, we found a significant induction of the ectodermal marker *TUBB3* (fold change = 3.31), the mesodermal marker *ACTA2* (fold change = 23.9) and the endodermal marker *AFP* (fold change = 1,319) in both 47XXY-iPSCs and 46XY-iPSCs as demonstrated by quantitative RT-PCR analysis (Figure 2B). Immunofluorescence analysis revealed that these iPSC lines differentiated in derivatives of all embryonic germ layers upon 3 weeks of spontaneous *in vitro* differentiation, as detected by the expression of the ectodermal marker β3-tubulin, the mesodermal marker α-SMA and the endodermal marker AFP (Figure 2C).

### iPSC Capacity to Differentiate Into Germ Cell Lineage

After 21 days of spontaneous *in vitro* differentiation, between 1 and 3.4% of cells derived from 47XXY-iPSCs and 46XY-iPSCs expressed VASA, a protein primarily found in germ cells (Figure 2D). Quantitative RT-PCR analysis revealed a significant decrease (fold change = 0.06) in the expression of *DAZL*, a very early marker of germ cells whereas the expression of *TEKT1* was significantly increased (fold change = 20.99) in cells derived from both iPSCs after 21 days of spontaneous differentiation. No difference was observed between cell lines (Figure 2E).

**FIGURE 3:**
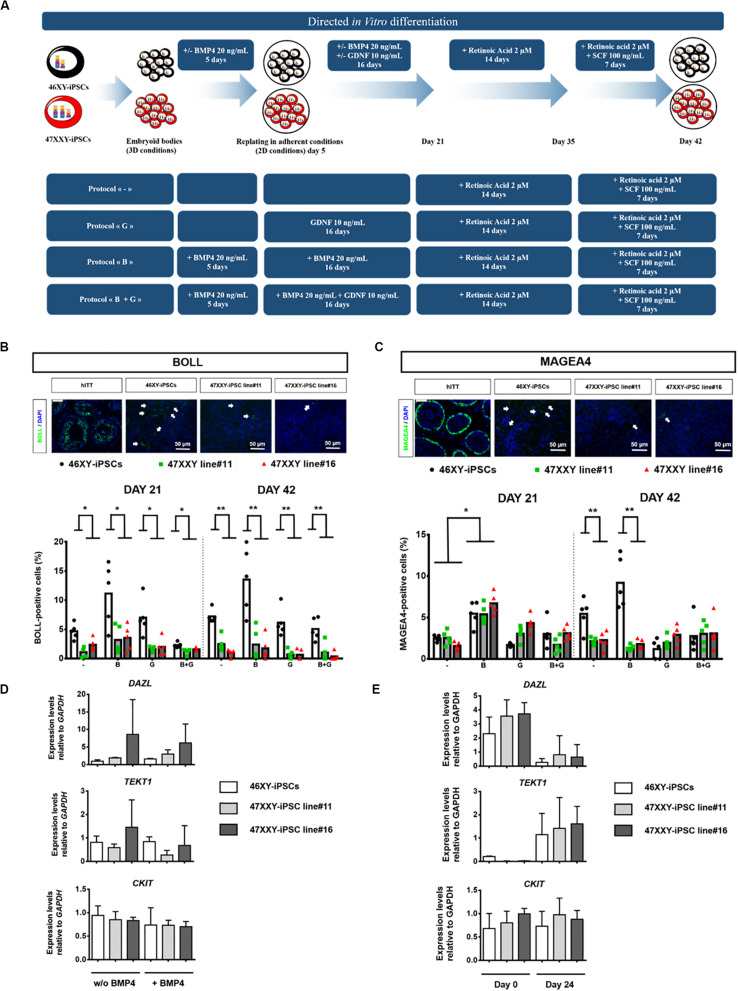
Directed *in vitro* differentiation of 46XX-iPSCs and 47XXY-iPSCs into germ cell lineage. **(A)** Schematic representation of the 4 different protocols used for iPSC differentiation into germ cell lineage. These protocols differed only in the first steps of the differentiation; with or without 20 ng/mL BMP4 in the 5 first days of differentiation and the presence or not of 10 ng/mL GDNF between day 5 and day 21 of differentiation. The last step of the protocols is identical with the presence of 2 μM retinoic acid between day 21 and day 35 and the presence of 2 μM retinoic acid + 100 ng/mL SCF between day 35 and day 42. **(B)** Immunofluorescence staining of 46XY-iPSC- and 47XXY-iPSC-derived cells for the germ cell marker BOLL. Representative images of 46XY-iPSC- and 47XXY-iPSC-derived cells at day 42 are represented. Human immature testicular tissue (hITT) was used as a positive control for BOLL expression (scale = 50 μM). Quantitative analysis of the proportion of BOLL positive cells for each protocol at day 21 and day 42 of germ cell differentiation. Data are represented as variance around mean, ^∗^*p* < 0.05 and ^∗∗^*p* < 0.01 between 46XY-iPSCs and 47XXY-iPSC line#11 and 47XXY-iPSC line#16 at day 21 and at day 42 from *n* = 5 independent experiments. **(C)** Immunofluorescence staining of 46XY-iPSC- and 47XXY-iPSC-derived cells for the germ cell marker MAGEA4. Representative images of 46XY-iPSC- and 47XXY-iPSC-derived cells at day 42 are represented. Human immature testicular tissue (hITT) was used as a positive control for MAGEA4 expression (scale = 50 μM). Quantitative analysis of the proportion of MAGEA4 positive cells for each protocol at day 21 and day 42 of germ cell differentiation. Data are represented as variance around mean, ^∗^*p* < 0.05 and ^∗∗^*p* < 0.01 between 46XY-iPSCs and 47XXY-iPSC line#11 and 47XXY-iPSC line#16 at day 21 and day 42 from *n* = 5 independent experiments. **(D)** Quantitative RT-PCR analysis of *DAZL*, *TEKT1*, and *CKIT* upon germ cell induction with or without 20 ng/mL BMP4 in the 5 first days. Expression levels are expressed relative to *GAPDH*. Data are represented from *n* = 3 independent experiments. **(E)** Quantitative RT-PCR analysis of *DAZL*, *TEKT1*, and *CKIT* upon germ cell differentiation of 46XY-iPSC and 47XXY-iPSC lines at day 0 and day 21. Expression levels are expressed relative to *GAPDH*. Data are represented from *n* = 3 independent experiments.

Given the critical roles of bone morphogenetic protein 4 (BMP4), glial-derived neurotrophic factor (GDNF), retinoic acid (as a meiosis-inducing factor) and stem cell factor (SCF) on germ cell differentiation (Kee et al., 2006; West et al., 2009; Spinnler et al., 2010; Singh et al., 2017; Teletin et al., 2017; Li et al., 2020; Mahabadi et al., 2020a,b), we tested different combinations of these factors to enhance germ cell differentiation of iPSCs as outlined in Figure 3A. After 21 days of differentiation, we observed a 2.4-fold increase of BOLL positive cells and a 2-fold increase of MAGEA4-positive cells when 46XY-iPSCs were treated with BMP4 compared to control conditions, although this increase was less marked after 42 days of differentiation: 1.9- and 1.7-fold increase for BOLL-positive cells and MAGEA4-positive cells, respectively (Figure 3B). After 42 days of differentiation, the proportion of MAGEA4 positive cells and BOLL positive cells had a tendency to be higher in BMP4-treated compared to control conditions but failed to reach significance (Figures 3B,C). Approximately, 13.55 and 9.2% of cells derived from 46XY-iPSCs expressed BOLL and MAGEA4, respectively (Figures 3B,C). Of note, upon this BMP4-treated protocol, we also found trophectoderm, mesoderm and endoderm derivatives as revealed by immunofluorescence staining and RT-PCR analysis (Supplementary Figure S2). To test whether the greater proportion of BOLL and MAGEA4 positives at day 21 in BMP4-treated compared to control conditions is due to differences in early germ cell induction, we further analyzed the expression of *DAZL*, *TEKT1*, and *CKIT* at day 5 of germ cell differentiation by RT-PCR (Figure 3D). No difference in the expression of these genes was found in 46XY-iPSC-derived cells regardless the presence or not of BMP4 (Figure 3D). However, in the presence of BMP4, we found a down regulation of *DAZL* and an upregulation of *TEKT1* between day 0 and day 21 of germ cell differentiation (Figure 3E).

### Reduced Ability of 47XXY-iPSCs to Differentiate Into Germ Cell Lineage

**FIGURE 4:**
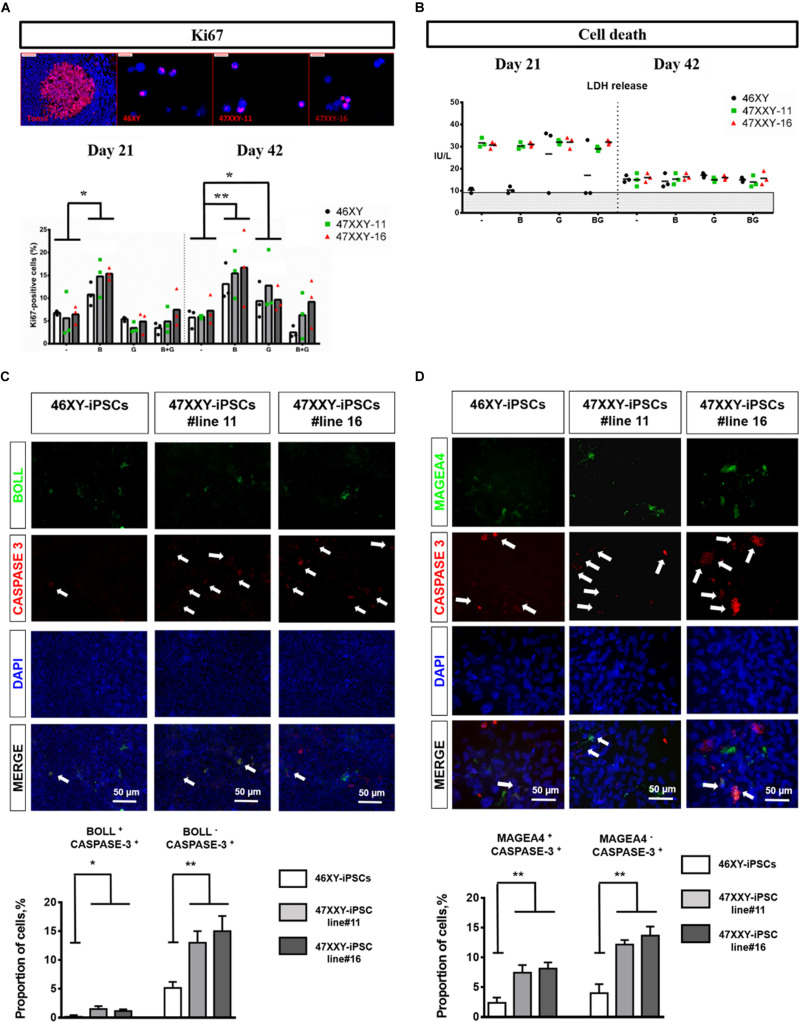
Cell proliferation and cell death analysis of 46XX-iPSCs and 47XXY-iPSCs upon germ cell differentiation. **(A)** Cell proliferation analysis by Ki-67 staining of 46XY-iPSC- and 47XXY-iPSC-derived cells for each protocol after day 21 and day 42 of differentiation. **(B)** Cell death analysis by LDH release by 46XY-iPSC- and 47XXY-iPSC-derived cells for each protocol after day 21 and day 42 of differentiation. Data are represented as variance around mean, ^∗^*p* < 0.05 and ^∗∗^*p* < 0.01 between 46XY-iPSCs and 47XXY-iPSC line#11 and 47XXY-iPSC line#16 at day 21 and day 42, from *n* = 3 independent experiments. **(C)** Immunofluorescence co-staining of 46XY-iPSC- and 47XXY-iPSC-derived cells for the germ cell marker BOLL and the marker of apoptosis cleaved caspase-3. Quantitative analysis of the proportion of BOLL and cleaved caspase-3 double positive cells and the proportion of BOLL negative and cleaved caspase-3 positive cells at day 21 of germ cell differentiation. **(D)** Immunofluorescence staining of 46XY-iPSC- and 47XXY-iPSC-derived cells for the germ cell marker MAGEA4 and the marker of apoptosis cleaved caspase-3. Quantitative analysis of the proportion of MAGEA4 and cleaved caspase-3 double positive cells and the proportion of BOLL negative and cleaved caspase-3 positive cells, at day 21 of germ cell differentiation. Data are represented from *n* = 3 independent experiments.

When 46XY-iPSCs and 47XXY-iPSCs were induced to differentiate into germ cell lineage, we found a reduced proportion of BOLL positive cells at day 21 in 47XXY-iPSC-derived cells (Figure 3B). By contrast, no difference was found for the proportion of MAGEA4 positive cells at this earlier step of differentiation (Figure 3C). Next, we assessed whether the reduced ability of 47XXY-iPSCs to generate BOLL positive cells is due to proliferation failure and/or increased cell death of 47XXY-iPSC-derived cells. As shown in Figure 4A, the proportion of Ki-67 positive cells was similar between 47XXY-iPSC-derived cells and 46XY-iPSC-derived cells at day 21, consistent with similar proliferation properties of these cells (Figure 4A). However, an overall increase of LDH release was found in 47XXY-iPSC-derived cells compared with 46XY-iPSC-derived cells at day 21, indicating a higher cell death in 47XXY-iPSC-derived cells (Figure 4B and Supplementary Figure S3). Then, to test which cells derived from 47XXY-iPSCs are responsible for this increase of cell death, we performed co-staining with BOLL or MAGEA4 and cleaved caspase-3 antibodies of 47XXY-iPSC-derived cells at day 21 (Figures 4C,D). Importantly, we found a greater proportion of BOLL and cleaved caspase-3 double positive cells (Figure 4C) and of MAGEA4 and cleaved caspase-3 double positive cells (Figure 4D) upon germ cell differentiation of 47XXY-iPSCs compared to 46XY-iPSCs, consistent with an increase of apoptosis of these cells. Of note, we also found a higher proportion of BOLL negative (or MAGEA4 negative) that were also cleaved caspase-3 positive cells, indicating an overall increase of apoptosis of 47XXY-iPSC-derived cells (Figures 4C,D).

Moreover, further differentiation of these iPSCs until day 42 revealed a reduced proportion of BOLL positive cells at day 42 in 47XXY-iPSC-derived cells compared with 46XY-iPSCs (Figure 3B). In line with this, we found a significant decrease in the proportion of MAGEA4 positive cells at day 42 in 47XXY-iPSC-derived cells compared to 46XY-iPSCs in both BMP4-treated and control conditions (Figure 3B), although this decrease appeared more important in the BMP4-treated (∼4.2-fold decrease) than in control conditions (∼2.4-fold decrease) (Figure 3B). These effects were not associated with a proliferation deficit as similar proportions of Ki-67 positive cells were found between 47XXY-iPSC-derived cells and 46XY-iPSC-derived cells at day 42 (Figure 4A). Furthermore, these effects could not be attributed to cell death differences as similar LDH release was found in both iPSC-derived cells at day 42 (Figure 4A and Supplementary Figure S3).

## Discussion

A major challenge in the field of Klinefelter syndrome (KS) research has been to recapitulate the disease phenotype and to understand the cellular and molecular mechanisms by which the extra copy of X chromosome leads to KS abnormalities (Lanfranco et al., 2004; Gravholt et al., 2018). In order to recapitulate the disease *in vitro*, iPSCs have been generated from patients with KS (Ma et al., 2012; Shimizu et al., 2016; Panula et al., 2019). However, to the best of our knowledge our study is the first investigating the differentiation potentials of iPSCs derived from a patient with KS (47XXY-iPSCs) into germ cell lineage using a directed differentiation protocol.

Several observations and findings arise from the current study investigating the abilities of 47XXY-iPSCs to differentiate into germ cell lineage when compared with 46XY-iPSCs. Consistent with previous reports (Clark et al., 2004; Chen et al., 2006; Panula et al., 2010), spontaneous *in vitro* differentiation of iPSCs as embryoïd bodies resulted in limited proportions of cells differentiated into germ like cells (between 1 and 3.4%). Therefore, in order to enhance germ cell differentiation of iPSCs, we used combinations of several factors, such as bone morphogenetic protein 4 (BMP4), glial-derived neurotrophic factor (GDNF), retinoic acid (as a meiosis-inducing factor), and stem cell factor (SCF) influencing germ cell differentiation (Kee et al., 2006; West et al., 2009; Spinnler et al., 2010; Singh et al., 2017; Teletin et al., 2017; Li et al., 2020; Mahabadi et al., 2020a,b), as outlined in Figure 3A. First, we found that certain key combinations of these factors increased germ cell differentiation compared to spontaneous *in vitro* differentiation protocol. This directed differentiation protocol recapitulates the main steps of germ cell development with the first 3 weeks devoted to obtain a germline and the last 3 weeks that were common across the protocols (2 μM retinoic acid for the first 2 weeks and 2 μM retinoic acid + SCF 100 ng/mL for the last week) to reach the first stages of post-meiotic development. Among the combinations used in our protocols, addition of BMP4 in the early steps of differentiation (the first 21 days) led to a ∼2-fold increase proportion of BOLL positive cells at day 21 and day 42. Similar results were found for MAGEA4 positive cells at day 21, but at day 42 the greater proportion of MAGEA4 positive cells in the presence of BMP4 failed to reach significance. Thus, we did not find any effect of BMP4 addition on gene expression of *VASA*, *CKIT*, and *DAZ1* after 5 days of differentiation. Conflicting results have been published regarding the requirement of BMP4 to start germ cell differentiation (Kee et al., 2006; West et al., 2009; Panula et al., 2010; Eguizabal et al., 2011). The concentrations of BMP4 used or the presence of co-culture systems may account for these discrepancies.

Given that GDNF is a key player of cell fate decision of spermatogonial cells regulating spermatogonial self-renewal and differentiation (Meng et al., 2000; Naughton et al., 2006; Oatley and Brinster, 2008), we tested whether addition of GDNF between day 5 and day 21 improved germ cell differentiation. However, neither the addition of GDNF nor the combination of BMP4 and GDNF improved the proportion of MAGEA4 or BOLL positive cells. We found in particular that the addition of GDNF did not improve proliferation but instead increased cytotoxicity in iPSC-derived cells. In line with these results, Meng and collaborators have shown that mice overexpressing *GDNF* exhibited an increase of GDNF production by Sertoli cells leading to the accumulation of undifferentiated spermatogonia by favoring self-renewing instead of differentiation (Meng et al., 2000). Whether the concentration of GDNF used in our study favors self-renewing instead of differentiation of iPSC-derived cells deserves further investigations.

Collectively, our results suggest an improved germ cell differentiation protocol for iPSCs than spontaneous *in vitro* differentiation. Consistent with previous reports, *DAZL* was down-regulated after 21 days of differentiation (Clark et al., 2004; Geijsen et al., 2004). This is of special interest as *DAZL* encodes RNA binding proteins required for germ cell development in several species and is considered as an early marker of mouse and human germ cell development (Ruggiu et al., 1997; Clark et al., 2004; Geijsen et al., 2004). As expected for efficient germ cell differentiation, after 21 days of germ cell differentiation, we found an upregulation of *TEKT1*, which is a specific marker of germ cells during their later stages of migration when they enter the gonads and progress through meiosis and gamete morphogenesis (Larsson et al., 2000; Clark et al., 2004). Finally, after 42 days of differentiation, approximately 9.2% of the cells were positive for MAGEA4, a pre-spermatogonia marker and 13.55% of the cells were positive for BOLL, a protein required for the germ cell development and spermatogenesis. Of note, we compared our protocol to the one recently established by Zhao and colleagues (Zhao et al., 2018). Importantly, after 21 days of germ cell differentiation, this latter protocol gives rise to ∼23.2% of BOLL positive cells and ∼22.8% of MAGEA4 positive cells (Supplementary Figures S4, S5), consistent with a better efficiency of this protocol to generate germ cells from iPSCs. Whether this increased efficiency can be maintained in long term culture remains to be determined.

Another major finding of this study is the reduced ability of 47XXY-iPSCs to differentiate into germ cell lineage compared to 46XY-iPSCs. We found in particular a reduced proportion of MAGEA4 positive cells after 42 days of differentiation of 47XXY-iPSCs regardless of the presence or not of BMP4 in the first 21 days of differentiation. In line with this, we found a reduced proportion of BOLL positive cells after 42 days of differentiation of 47XXY-iPSCs. This decrease of MAGEA4 positive cells and BOLL positive cells could not be attributed to proliferation failure but rather and most likely to an increase of cell death at day 42. This increase of cell death was observed regardless of the addition of BMP4 during the early steps of differentiation. Our results are in contrast with those of Ma and colleagues as they found an aberrant transcriptome of 47XXY-iPSCs at the undifferentiated state but exhibit similar expression of germ cell lineage markers as 46XY-iPSCs (Ma et al., 2012). In accordance with our results, several studies have pointed out an increase of cell death affecting germ cells upon differentiation and maturation as a main mechanism leading to KS testis dysfunction (Aksglæde et al., 2005; D’Aurora et al., 2015, 2017). The molecular mechanisms by which the extra copy of X chromosome leads to this increase of apoptosis in germ cells from patients with KS are poorly understood. The main obstacle for genotype-phenotype correlation studies for KS is that approximately 15% of X chromosomal genes escapes X chromosomal inactivation and 10% of X chromosomal genes shows tissue specific expression (Carrel and Willard, 1999, 2005; Balaton et al., 2015). Some X chromosomal genes have been proposed to contribute directly or indirectly to the KS phenotype including *SHOX* (Ottesen et al., 2010), *XIST* (Ma et al., 2012; Belling et al., 2017; Winge et al., 2017; Panula et al., 2019), *AKAP17A* (Belling et al., 2017; Winge et al., 2017; Skakkebæk et al., 2018; Panula et al., 2019), *SLC25A6* (Zitzmann et al., 2015; Belling et al., 2017; Skakkebæk et al., 2018; Panula et al., 2019), *HDHD1* (Zitzmann et al., 2015; Panula et al., 2019), *NLGN4X* (Winge et al., 2017; Panula et al., 2019), *PLCXD1* (Belling et al., 2017; Panula et al., 2019), and *LDOC1* (Salemi et al., 2016). Among those genes, *SHOX* has been shown to trigger the lysosomial pathway of apoptosis (Hristov et al., 2013) while *LDOC1* has been shown to inhibit cell proliferation and promote apoptosis (Zhao et al., 2015). Whether these genes contribute to the greater apoptosis of 47XXY-iPSC-derived cells upon germ cell differentiation deserves further investigations.

## Conclusion

In conclusion, the generation of iPSCs from patients with KS provide an innovative model to study the effect of the supernumerary X chromosome on KS features. Importantly, 47XXY-iPSCs exhibited a reduced ability to differentiate into germ cells compared with 46XY-iPSCs. Our results further emphasize that this defect is more related to an increased cell death at day 21 of differentiation than a proliferation deficit of 47XXY-iPSC-derived cells. Although post-meiotic germ cell differentiation from 47XXY-iPSCs was shown in our study, another aspect that was not investigated but can be of interest for further investigations is the analysis of the ploidy of the germ cells differentiated from 47XXY-iPSCs. This could help to close the debate on the ability of KS germ cells to enter meiosis given that it was suggested that only diploid XY germ cells are competent to engage in meiosis in patients with KS and mouse model of KS (Mroz et al., 1999; Sciurano et al., 2009).

## Data Availability Statement

All datasets generated for this study are included in the article/Supplementary Material, further inquiries can be directed to the corresponding author.

## Ethics Statement

The animal study was reviewed and approved by the Ethics Review Board and the Committee on Animal Research of the Catholic University of Louvain.

## Author Contributions

OB contributed to fund raising, designed and performed research, data analysis and interpretation, figure preparation, and manuscript writing. YH performed some of the research data analysis and interpretation, figure preparation, and manuscript writing. MG participated to critical review of the manuscript. JA performed data analysis. CC performed some of the research. AF performed final approval of the manuscript. CW designed research, contributed to data analysis and interpretation, manuscript writing, fund raising, and final approval of the manuscript.

## Conflict of Interest

The authors declare that the research was conducted in the absence of any commercial or financial relationships that could be construed as a potential conflict of interest.
