# Supplementary material for: Modeling Klinefelter Syndrome Using Induced Pluripotent Stem Cells Reveals Impaired Germ Cell Differentiation
Source: Front Cell Dev Biol. 2020 Oct 7;8:567454. doi: 10.3389/fcell.2020.567454 (PMC7575714; doi:10.3389/fcell.2020.567454)
Supplement: Supplementary file 1 [file Data_Sheet_1.PDF]

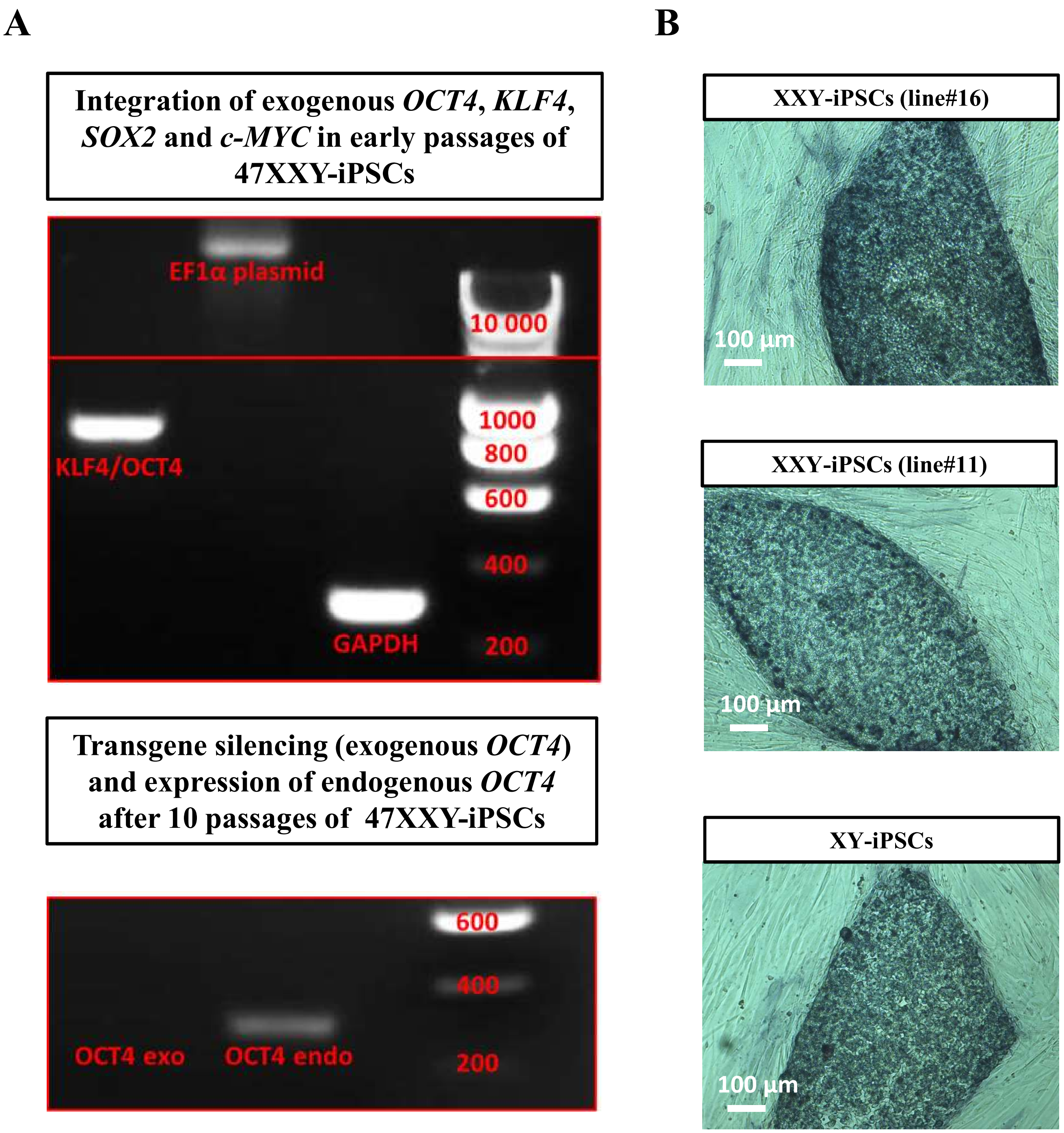

Supporting Information Figure 1. Additional controls validating the successful reprogramming and generation of 47XXY-iPSCs and 46XY-iPSCs.
(**A**) Integration of exogenous *OCT4*, *KLF4*, *SOX2* and *c-MYC* in early passages of 47XXY-iPSCs and Transgene silencing (exogenous *OCT4*) and expression of endogenous *OCT4* after 10 passages of 47XXY-iPSCs
(B) Alkaline phosphatase activity staining of 47XXY-iPSCs and 46XY-iPSCs.

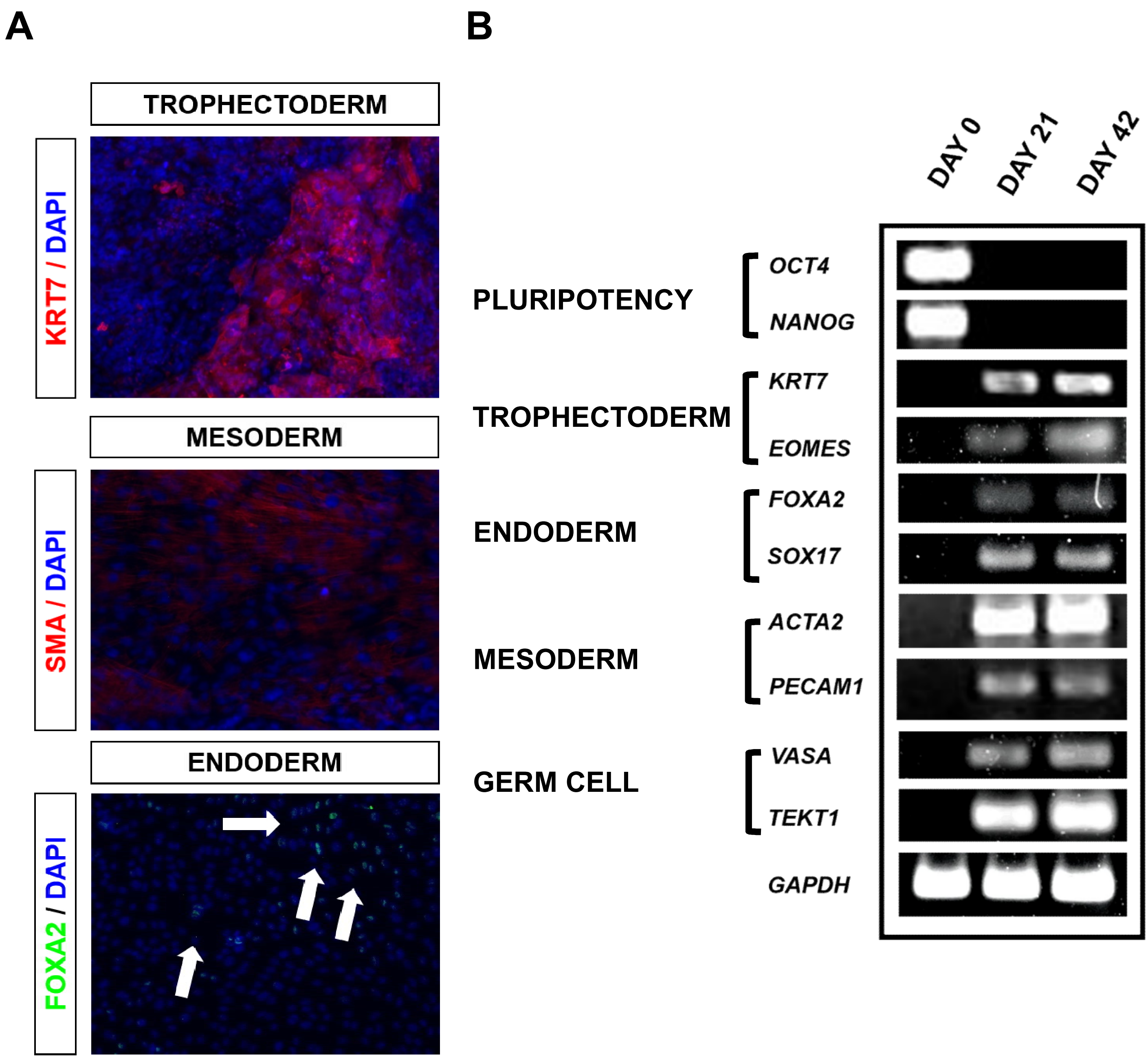

Supporting Information Figure 2. Additional controls showing the presence of cells from trophectoderm, mesoderm and endoderm lineage upon germ cell differentiation of 47XY-iPSCs.

(**A**) Immunofluorescence staining analysis showing the presence of KRT7 positive, SMA positive and FOXA2 positive cells upon germ cell differentiation of 47XY-iPSCs (day 42).

(B) RT-PCR analysis for markers of pluripotency (*OCT4* and *NANOG*), trophectoderm (*KRT7* and *EOMES*), endoderm (*FOXA2* and *SOX17*), mesoderm (*ACTA2* and *PECAM1*) and germ cells (*VASA* and *TEKT1*) following germ cell differentiation of 47XY-iPSCs at day 0, 21 and 42.

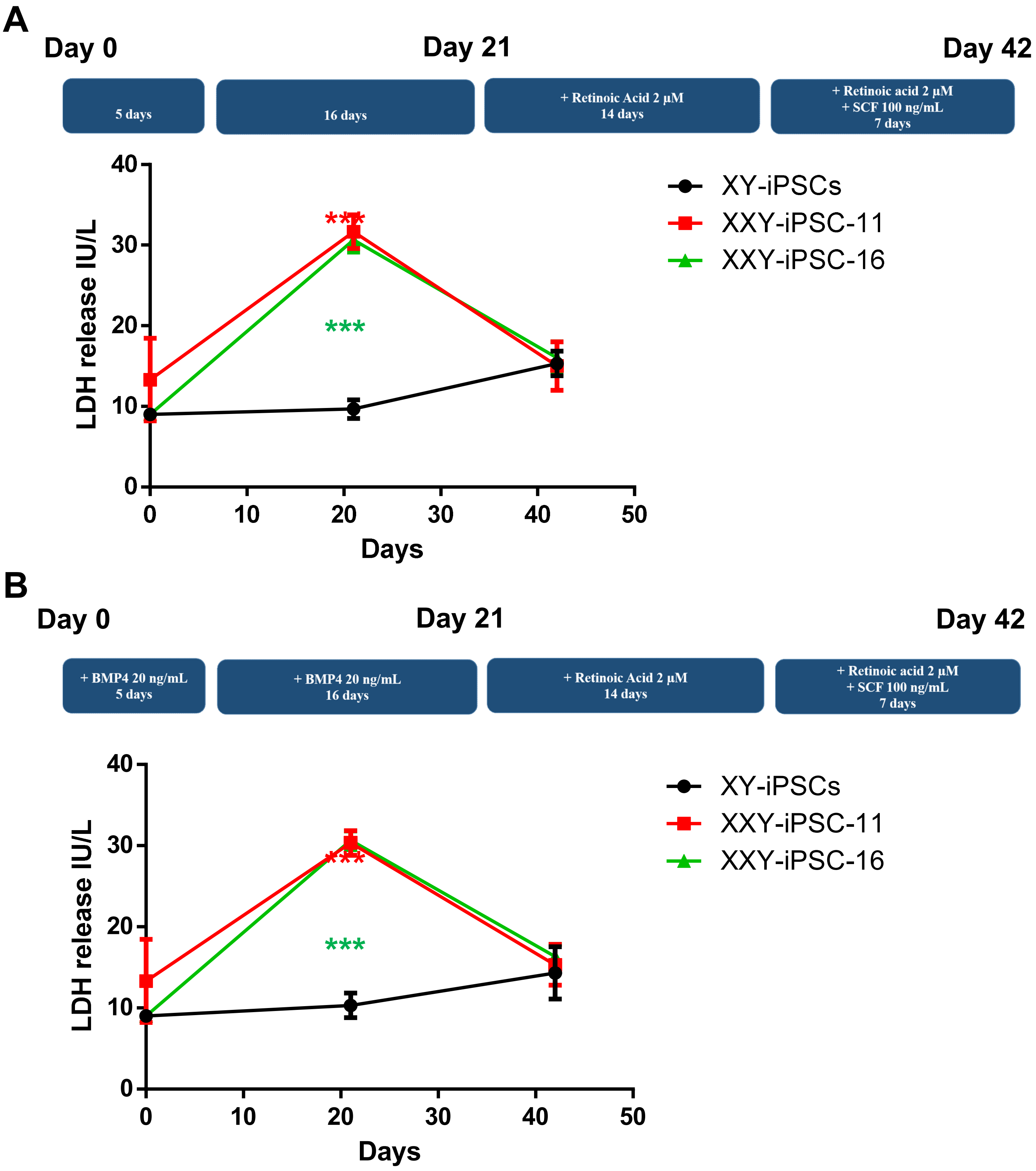

Supporting Information Figure 3. Kinetics of LDH release at day 0, 21 and 42 upon germ cell differentiation of 46XY-iPSCs and 47XXY-iPSCs in the absence of BMP4 (in A) or in the presence of BMP4 (in B) in the protocol.

A

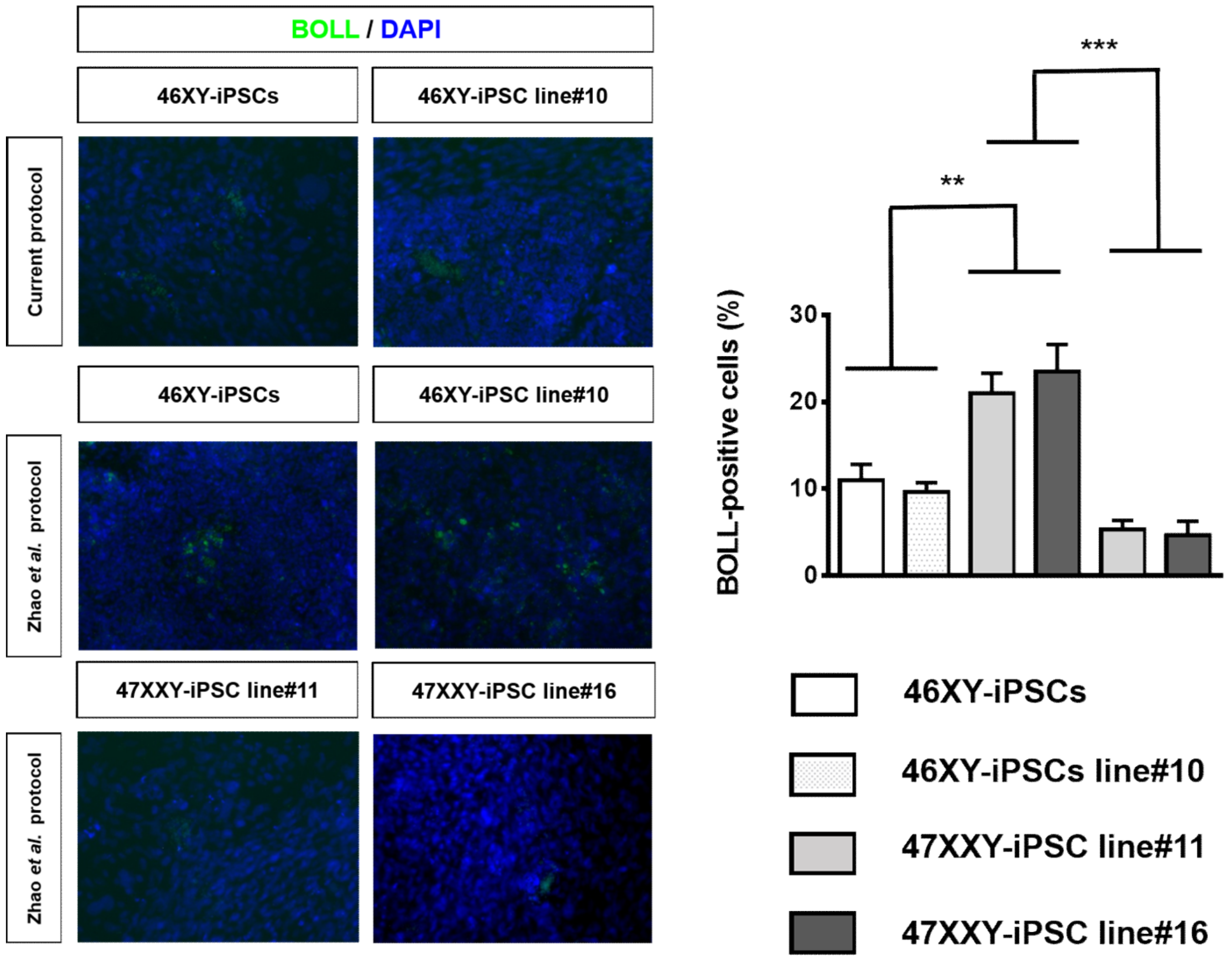

Supporting Information Figure 4. Comparative efficiency of Zhao and colleagues protocol with our protocol using BMP4 to generate germ cells. .
Immunofluorescence staining of 46XY-iPSC- and 47XXY-iPSC-derived cells for the marker of germ cells BOLL. Representative images of 46XY-iPSC- and 47XXY-iPSC-derived cells at day 21 are represented. Quantitative analysis of the proportion of BOLL positive cells for each protocol at day 21 of germ cell differentiation. Data are represented from n=3 independent experiments.

A

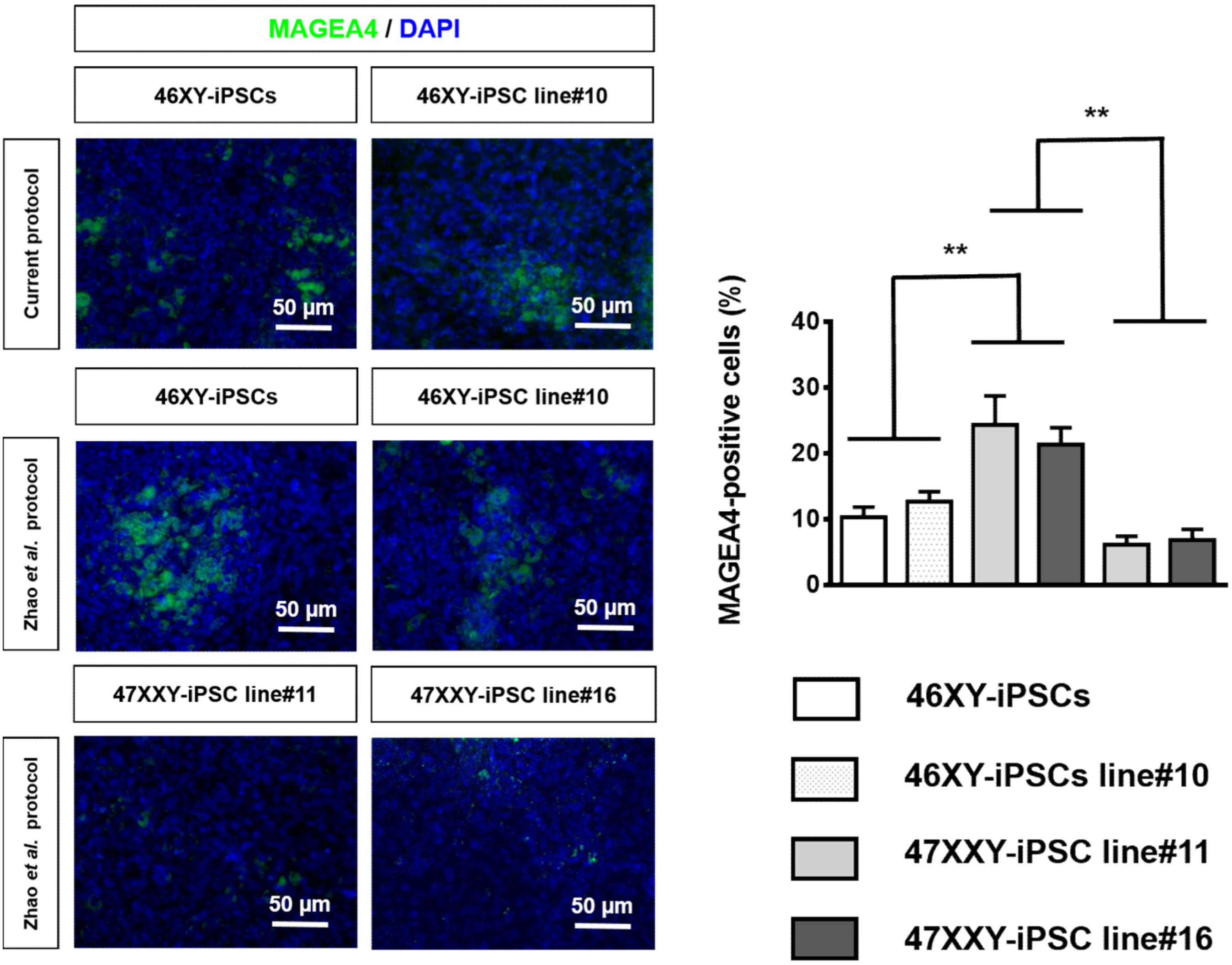

Supporting Information Figure 5. Comparative efficiency of Zhao and colleagues protocol with our protocol using BMP4 to generate germ cells.
Immunofluorescence staining of 46XY-iPSC- and 47XXY-iPSC-derived cells for the marker of germ cells MAGEA4. Representative images of 46XY-iPSC- and 47XXY-iPSC-derived cells at day 21 are represented. Quantitative analysis of the proportion of MAGEA4 positive cells for each protocol at day 21 of germ cell differentiation. Data are represented from n=3 independent experiments.

PROTEIN	DILUTION	CLONE	COMPANY Cat#
**OCT3/4**	1/250	Rabbit polyclonal	Abcam, Cat# ab18976,
**NANOG**	1/250	H-155 rabbit polyclonal	Santa Cruz, Cat# Sc-33759,
**TRA1-60**	1/250	Mouse monoclonal	Santa Cruz, Cat# Sc-21705,
**B3-TUBULIN**	1/1000	Rabbit polyclonal	Covance, Cat# PRB-435P,
**α-SMOOTH MUSCLE ACTIN**	1/400	Mouse moncoclonal	Sigma, Cat# A2547,
**α-FETOPROTEIN**	1/250	C3 mouse monoclonal	Santa Cruz, Cat# Sc-8399,
**VASA**	1/100		Abcam, Cat# ab27591,
**MAGEA4**	1/100	Mouse monoclonal	Purified from hybridoma 57B and kindly provided by Giulio Spagnoli MD, University of Basel, Switzerland
**Ki-67**	1/100	Rabbit monoclonal	Abcam, Cat# ab92353,
**CLEAVED CASPASE-3**	1/250<br>1/400	3D9 mouse monoclonal<br>Rabbit polyclonal	Millipore, Cat#10753<br>Cell signalling, Cat#9661,
**DAZ1**	1/50	3G10 mouse monoclonal	Abcam, Cat# ab115548,
**BOLL**	1/100	Rabbit polyclonal	Sigma, Cat# HPA018678,

Supporting Information Table 1. Antibodies used in the study

Gene	Forward	Reverse
GAPDH	AGCCACATCGCTCAGACACC	GTACTCAGCGGCCAGCATCG
OCT4 endo	GCCTTCCTTCCCCATGGC	CCTCAAAATCCTCTCGTTGT
OCT4 exo	TCAAGCCTCAGACAGTGGTTC	CCTCAAAATCCTCTCGTTGT
STEMCCA OCT3/4-KLF4 junction	ACCATCTGTCGCTTCGAGGCC	GGCTAGGAGGGCCGGGTTGTT
LIN28	AGCCATATGGTAGCCTCATGTCCGC	TCAATTCTGTGCCTCCGGGAGCAGGGTAGG
KLF4	TTCCTGCATGCCAGAGGAGCCC	AATGTATCGAAGGTGCTCAAG

Supporting Information Table 2. Primer sequences for non-quantitative RT-PCR

Gene	Forward	Reverse
GAPDH	TGCACCACCAACTGCTTAGC	GGCATGGACTGTGGTCATGAG
AFP	TTGACTGCAATTGAGAAACCCA	AAGGCAGGTAGCTGGTTTTCTAAA
DAZL	ATGTTAGGATGGATGAAACTGAGATTA	CCATGGAAATTTATCTGTGATTCTACT
NANOG	TGAAGCTCAGCTACAAACAG	TGGTGGTAGGAAGAGTAAAG
OCT4	AGTGCCCGAAACCCACACTG	ACCACACTCGGACCACATCCT
CKIT	ATTCTCAGACTTGGGATAATC	CAGGCAACGTTGACTATCAGT
ACTA2	AATACTCTGTCTGGATCGGTGGCT	ACGAGTCAGAGCTTTGGCTAGGAA
TEKT1	TGGTGCTGGAGAAGTCCAACCAGTCTT	AAGTACAATCTTGAGAAGGATTTGAA
TUBB3	CAACAGCACGGCCATCCAGG	CTTGGGGCCCTGGGCCTCCGA
VASA	AGAAAGTAGTGATACTCAAGGACCAA	TGACAGAGATTAGCTTCTTCAAAAGT
EOMES	CGGCCTCTGTGGCTCAAA	AAGGAAACATGCGCCTGC
KRT7	GATGCTGCCTACATGAGCAA	GATGATGCCGTCCAGGTC
FOXA2	TGCACTCGGCTTCCAGTATG	GGAGGAGTAGCCCTCGG
SOX17	GTGAATCTCCCCGACAGC	TGTTTTGGGACACATTCAAAGC
PECAM1	AACGGAAGGCTCCCTTGATG	TAAGAACCGGCAGCTTAGCC

Supporting Information Table 3. Primer sequences for non-quantitative and quantitative RT-PCR
